# Placental Pathology Associated with Household Air Pollution in a Cohort of Pregnant Women from Dar es Salaam, Tanzania

**DOI:** 10.1289/EHP256

**Published:** 2016-06-10

**Authors:** Blair J. Wylie, Emmanuel Matechi, Yahya Kishashu, Wafaie Fawzi, Zul Premji, Brent A. Coull, Russ Hauser, Majid Ezzati, Drucilla J. Roberts

**Affiliations:** 1 Department of Obstetrics/Gynecology, Massachusetts General Hospital, Boston, Massachusetts, USA; 2 Department of Environmental Health, Harvard T.H. Chan School of Public Health, Boston, Massachusetts, USA; 3 Harvard Medical School, Boston, Massachusetts, USA; 4 African Academy for Public Health, Dar es Salaam, Tanzania; 5 Muhimbili University of Health and Allied Sciences, Dar es Salaam, Tanzania; 6 Department of Global Health and Population,; 7 Department of Biostatistics, and; 8 Department of Epidemiology, Harvard T.H. Chan School of Public Health, Boston, Massachusetts, USA; 9 MRC-PHE Centre for Environment and Health, School of Public Health, Imperial College London, London, United Kingdom; 10 Department of Pathology, Massachusetts General Hospital, Boston, Massachusetts, USA

## Abstract

**Background:**

Smoke from the burning of biomass fuels has been linked with adverse pregnancy outcomes such as low birth weight, stillbirth, and prematurity.

**Objective:**

To identify potential underlying mechanisms of adverse perinatal outcomes, we explored the association of placental pathology with household air pollution in pregnant women from urban/periurban Tanzania who cook predominantly with charcoal.

**Methods:**

Between 2011 and 2013, we measured personal exposures to fine particulate matter (PM2.5) and carbon monoxide (CO) over 72 hr among a cohort of Tanzanian pregnant women. Placentas were collected after delivery for examination. Placental pathologies of inflammatory, hypoxic, ischemic/hypertensive, infectious and thrombotic etiologies were diagnosed, blinded to exposure levels. Using multiple logistic regression, we explored the association of PM2.5 and CO exposure with placental pathology.

**Results:**

One hundred sixteen women had personal air exposure measurements and placental histopathology available for analysis. PM2.5 and CO exposures were moderate [geometric means (GSD) were 40.5 μg/m3 (17.3) and 2.21 ppm (1.47) respectively]; 88.6% of PM2.5 measurements exceeded World Health Organization air quality guidelines. We observed an increase in the odds (per 1-unit increase in exposure on the ln-scale) of fetal thrombotic vasculopathy (FTV) both with increasing PM2.5 [adjusted odds ratio (aOR) = 5.5; 95% CI: 1.1, 26.8] and CO measurements (aOR = 2.5; 95% CI: 1.0, 6.4) in adjusted models only. FTV also was more common among pregnancies complicated by stillbirth or low birth weight.

**Conclusions:**

Fetal thrombosis may contribute to the adverse outcomes associated with household air pollution from cook stoves during pregnancy. Larger studies are necessary for confirmation.

**Citation:**

Wylie BJ, Matechi E, Kishashu Y, Fawzi W, Premji Z, Coull BA, Hauser R, Ezzati M, Roberts D. 2017. Placental pathology associated with household air pollution in a cohort of pregnant women from Dar es Salaam, Tanzania. Environ Health Perspect 125:134–140; http://dx.doi.org/10.1289/EHP256

## Introduction

An estimated 40% of the world’s population relies on solid biomass fuels (wood, charcoal, crop residues) for cooking or heating (Bonjour et al. 2013). The smoke generated from the combustion of these fuels is a complex mixture of gases and fine particles including many known harmful pollutants, such as carbon monoxide (CO), suspended fine particulate matter (≤ 2.5 μm; PM_2.5_), nitrogen oxide, and polycyclic aromatic hydrocarbons. Household air pollution from biomass burning is now acknowledged as a major contributor to the global disease burden (Lim et al. 2012).

During pregnancy, exposure to biomass cooking smoke has been linked with a reduction in birth weight, an increase in stillbirth, and a rise in preterm births in a number of epidemiologic investigations (Amegah et al. 2014). Despite a growing evidence base of reproductive harm from household air pollution, methodological shortcomings remain, including challenges in exposure assessment and the potential for uncontrolled confounding. Pathological examination of placentas from pregnant women exposed to biomass cook smoke may identify lesions that are known to be associated with adverse pregnancy outcomes. Furthermore, specific lesions may shed light on the underlying pathophysiology and suggest targets for amelioration of risk. For these reasons, we aimed to characterize placental pathology associated with household air pollution among women cooking with biomass fuels in an African population.

**Figure 1:**
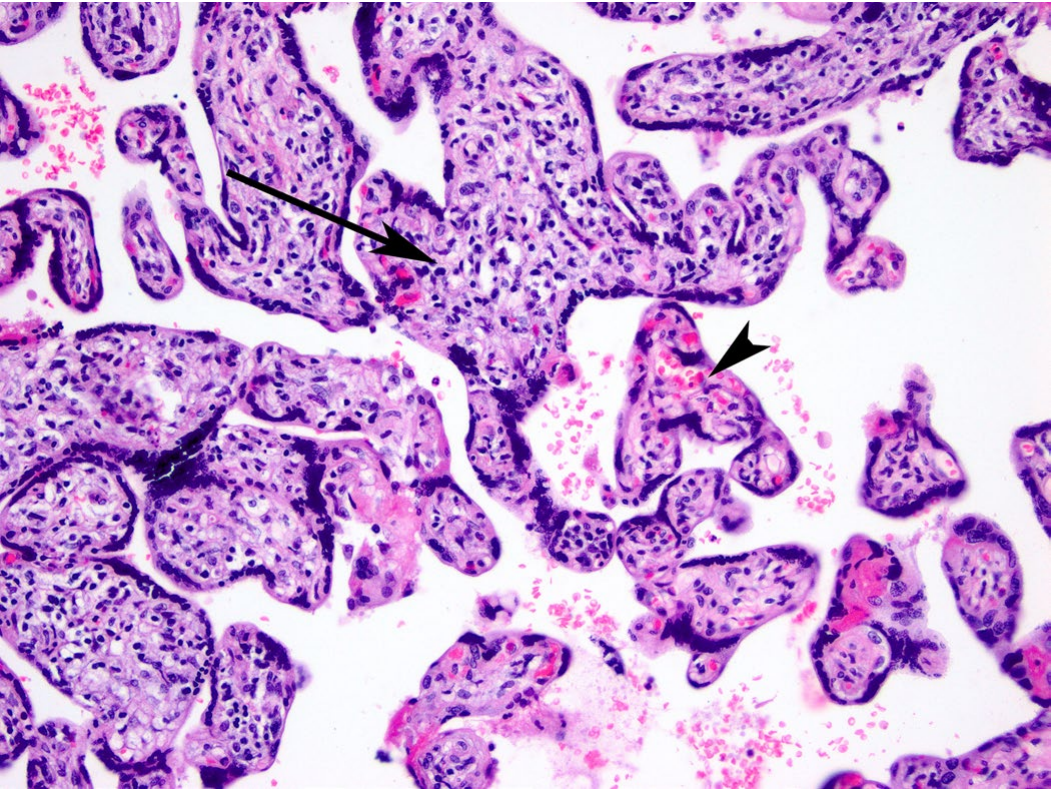
Representative sample of chronic villitis (long arrow) and normal non-inflamed villous (arrowhead). Note increased stromal cellularity and decreased vascularity in villi affected by chronic villitis. Magnification: 20×.

**Figure 2:**
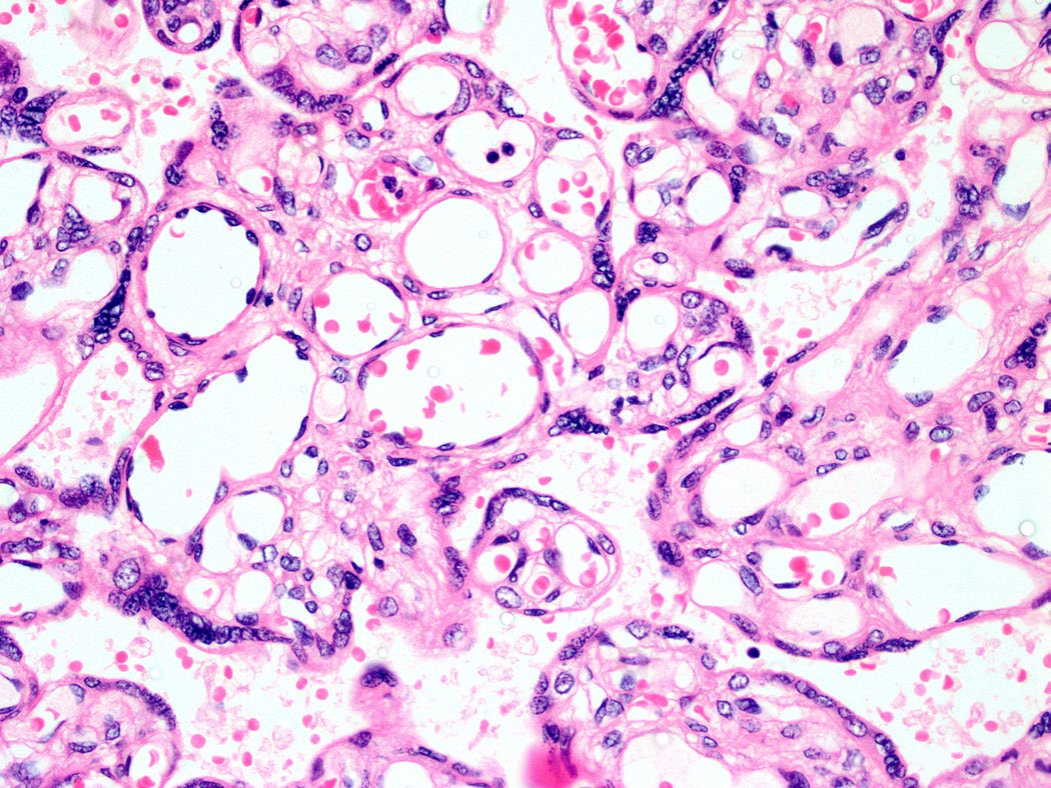
Representative sample of chorangiosis. Mature chorionic villi showing increased numbers of capillaries meeting diagnostic criteria for chorangiosis. Magnification: 40×.

We hypothesized that the placenta would demonstrate inflammatory lesions in the setting of high household air pollutant exposure, and specifically when levels of PM_2.5_ exposure are high. Cardiopulmonary researchers have demonstrated both that inhaled ultrafine particles can enter the circulation and that systemic measures of inflammation increase following exposure to air pollution (Donaldson et al. 2005; Scapellato and Lotti 2007). We posited that damage to the placenta may occur directly from exposure to circulating particles or secondary to systemic inflammation, either of which may result in any of the well-defined histologic placental chronic inflammatory lesions—chronic villitis (Figure 1), chronic chorioamnionitis, or intervillositis. Second, we hypothesized that biomass smoke exposed placentas would demonstrate hypoxic lesions, particularly as CO levels increase. CO impairs placental oxygen transport by increasing levels of carboxyhemoglobin, thereby displacing oxygen from hemoglobin and reducing its availability to the fetus (Soothill et al. 1996). The placental response in the setting of hypoxia is an increase in the surface area and vascularity of the villi (Altshuler 1984). This adaptive angiogenesis is manifest as chorangiosis (Figure 2), a common feature of tobacco-exposed placentas (Pfarrer et al. 1999).

## Methods

### Overview

Between January 2011 and May 2013, personal exposures to PM_2.5_ and CO were measured during the second or third trimester for a subset of pregnant women enrolled in the Prenatal Iron Supplements study (NCT01119612; http://clinicaltrials.gov/show/NCT01119612). This parent trial recruited only HIV-negative women in their first or second pregnancies living in an urban/periurban setting in and around Dar es Salaam, Tanzania (Etheredge et al. 2015). For our substudy on household air pollution, we approached only nonsmoking pregnant women who were the primary cooks in their household and were ≥ 15 years of age to participate in exposure measurement. Study subjects answered a number of questions specific to their cooking practices, including types of fuel used, hours spent cooking, stove design, cooking area ventilation, smoking habits of other household members, as well as exposure to other sources of pollution such as traffic, tobacco, incense, and the burning of rubbish. As part of the parent trial, standard operating procedures for gestational age assessment, birth measurements, and collection of the placenta were followed by trained research staff. Placental malaria was the primary outcome of the Prenatal Iron Supplements study, so trial procedures were optimized to encourage delivery at one of the study-affiliated facilities to enable collection of the placenta. Furthermore, the placentas were processed for histopathologic examination regardless of whether pregnancy complications occurred and therefore are more representative of the general population in this study.

Subjects were eligible for the analysis presented in this manuscript if a personal exposure measure of either PM_2.5_ or CO was successfully obtained during pregnancy and placental histopathology was available. The study protocol was approved by the institutional review boards of Muhimbili University of Health and Allied Sciences, the Harvard T.H. Chan School of Public Health, and Partners Healthcare (for Massachusetts General Hospital). Informed consent was obtained from all participants.

### Exposure Monitoring

CO was measured over 72 hr using passive diffusion tubes (Draeger Carbon Monoxide 50/a-D; Draeger USA) clipped to the woman’s clothing, and measurements of the length of color change were used to calculate an average concentration in parts per million (ppm). PM_2.5_ exposure was measured during the first and third 24 hr of the 72-hr sampling period using a portable pump worn by the subject. Filters were weighed on a Mettler Toledo MT5 microbalance after conditioning in a temperature- and humidity-controlled environment for at least 24 hr and statically discharged via a polonium source. All filter weights took into account correction for lab blanks. The two 24-hr PM_2.5_ mass concentrations were averaged to represent the personal PM_2.5_ exposure. Exposure measurements were conducted only once per subject during a single 72-hr period in either the second (12%) or third (88%) trimester of pregnancy. For additional details regarding CO and PM_2.5_ measurements, see Supplemental Material, “Details of carbon monoxide exposure measurement” and “Details of fine particulate matter exposure measurement.”

### Placental Sampling, Histopathologic Processing and Interpretation

Before the study began, research nurses received hands-on training and skills verification in placental sampling by a U.S. perinatal pathologist (D.J.R.). Similarly, study pathology technicians were trained and supervised by a U.S. perinatal pathologist (D.J.R.). Following birth, portions of the umbilical cord, membranes, and three full thickness sections of a prespecified size (approximately 3 cm^3^) from the placental disk were obtained by study nurses. Palpable or visible abnormalities of the placental disk were preferentially targeted for sampling. The cord and membranes were subsequently excised and the placenta weighed with an electronic scale to the nearest gram. All placental samples obtained were placed in 10% neutral buffered formalin and then transported to the pathology lab at Muhimbili University where they remained in fixation for at least 4 hr up to a maximum of 24 hr. Following fixation, samples were trimmed and routinely processed to produce hematoxylin/eosin-stained slides. All births occurred in one of the study hospitals. Study nurses were stationed at study facilities around the clock to complete placental sampling in a timely manner. Sampling occurred within the first hour after delivery for most subjects; if this could not be accomplished, placentas were placed in a facility refrigerator until sampling occurred.

Slides were shipped to Massachusetts General Hospital where they underwent histopathologic examination by an experienced perinatal pathologist (D.J.R.) who was blinded to details regarding cooking fuel and measured exposure levels. Between one and three parenchymal slides were available for review. Diagnoses were rendered using standard diagnostic criteria (Altshuler 1984; Chisholm and Heerema-McKenney 2015; Ogino and Redline 2000; Redline et al. 2003, 2004a, 2004b; Roberts 2008, 2013; Schwartz 2001) and coded using a standardized form. Diagnoses were made on the routine hematoxylin/eosin-stained slides; no special studies were obtained. Specific lesions were assigned into the categories of hypoxic, hypertensive/ischemic, inflammatory, infectious, and thrombotic as outlined in Table 1. Thrombotic lesions were further subclassified as fetal or maternal in origin. More than one diagnosis could be rendered for a given subject. Although our specific hypothesis was that the placenta would show adaptations related to inflammation or hypoxia, all other histopathologic findings were noted and compared with exposure data.

**Table 1:** Categorization of placental lesions.

Type	Lesion
Hypoxic	Chorangiosis
	Edema
Ischemic/hypertensive^*a*^	Infarcts
	Distal villous hypoplasia
	Decidual vasculopathy
	Villous agglutination
	Abruption
Inflammation (without infection)	Chronic villitis
	Intervillositis
	Chronic chorioamnionitis
Infection	Acute chorioamnionitis (maternal and/or fetal)
	Malaria
Thrombotic	
Maternal	Increased perivillous fibrin
	Massive perivillous fibrin distribution
	Maternal floor infarct
	Intervillous thrombi
	Subchorionic thrombus
Fetal	Fetal thrombotic vasculopathy
Other	Meconium
	Amniotic metaplasia (± clear cell)
	Calcifications
	Maternal sickling
	Villous dysmaturity
^***a***^Given the overlap of ischemic and hypertensive lesions, these were grouped together for analysis.	

Placental weights were compared against a United States reference standard (Pinar et al. 1996) as placental weight standards for Tanzania currently do not exist. Although the relevance of this standard to Tanzanian placentas is unclear, extremes of the standard should remain relevant. Placentas were therefore characterized as small for gestational age (< 10th percentile), large for gestational age (> 90th percentile), or appropriately sized. Gestational age was assigned in the parent trial by means of a postnatal new Ballard examination conducted by facility-based research staff within 24 hr following delivery (Ballard et al. 1991). The total of both the neurologic and external features scores was translated into gestational age in weeks using the published Ballard maturity-rating tables (Ballard et al. 1991).

### Data Analysis

Subjects were grouped into tertiles of exposure for both PM_2.5_ and CO to evaluate exposure–response associations. The Cochran Armitage trend test was used to evaluate whether the prevalence of placental lesions in a given category (hypoxic, ischemic/hypertensive, inflammatory, infectious, thrombotic-maternal, thrombotic-fetal) increased across exposure tertiles and two-sided *p*-values reported. If any cell count was < 5, an exact *p*-value was reported rather than the asymptotic approximation. Similar analyses were performed to compare the proportion of small for gestational age placental weights and large for gestational age placental weights across tertiles of PM_2.5_ and CO exposure.

Models were also fit using multiple logistic regression with exposure represented as a continuous predictor of placental lesion or placental weight categories. Exposure measurements for both PM_2.5_ and CO were natural log (ln)*–*transformed given the skews in measurement distributions. Candidate covariates were selected *a priori* and included maternal age (18–20, 21–25, ≥ 26 years), body mass index (< 18.5, 18.5–24.9, 25–29.9, ≥ 30 kg/m^2^), secondhand smoke exposure, season of exposure measurement (rainy vs. dry), and a household asset index (low: 0–5, medium: 6–8, high: 9–10) constructed after tallying household ownership of 10 items (car, generator, bicycle, sofa, television, radio, refrigerator, fan, electricity, and potable water). Additional covariates were considered including maternal hypertension, antenatal treatment for malaria, and tobacco use during pregnancy but were not included in the adjusted models given the low prevalence of these conditions and the consequent destabilization of effect estimates. No adjustments were made for multiple comparisons. Finally, we explored associations between each of the placental lesion categories and adverse pregnancy outcomes, specifically low birth weight (< 2,500 g) and stillbirth; unadjusted odds ratios (ORs) and 95% confidence intervals (CIs) were estimated using logistic regression for both stillbirth and low birth weight.

Analyses were performed separately for PM_2.5_ and CO exposures. Statistical analyses were performed using SAS software version 9.4 (SAS Institute Inc.).

## Results

Of the 239 primigravid or secundigravid women recruited for PM_2.5_ and CO exposure monitoring during pregnancy in the larger study, 116 had placental slides available for histopathologic review. All 116 had CO measurements successfully obtained. PM_2.5_ measurements were available for 79 of the 116 (see Figure S1). Exposure measurement occurred during 2011 and 2012 for all subjects except one who was recruited during 2013. Maternal characteristics, sociodemographics, cooking behaviors, kitchen characteristics, and other sources of household air pollution are summarized in Table 2. The women were recruited primarily from urban or periurban households in and around Dar es Salaam, Tanzania. One hundred ten of 116 (94.8%) were the primary cooks in their household, and the majority cooked three meals per day (74 of 116, 63.8%). Cooking areas were commonly shared with other families (54 of 116, 46.6%). Almost one-third of the cooking areas were located outdoors (37 of 116, 31.9%); this varied somewhat depending on the rain. Approximately half of the outdoor cooking areas were located under a roof and the remainder in the open air. For those cooking inside, a separate cooking area located in a different structure from the main house was used by about one-third of the women (34–37% depending on the season). Almost all indoor cooking spaces were ventilated by at least one window or an open door. The primary household fuel was charcoal in both the dry (93 of 116, 80.2%) and rainy seasons (62 of 116, 53.4%). Kerosene was the second most common fuel, with use increasing during the rainy season (46 of 116, 39.6%) compared with the dry season (12 of 116, 10.3%). Only two subjects cooked with gas or electricity, and < 10 households used wood as the primary fuel. When this cohort of 116 subjects was compared with the 239 subjects that constituted the larger study on exposure to household air pollutants during pregnancy, there were no significant differences identified with regard to any of the characteristics presented in Table 2 (data not shown).

**Table 2:** Maternal characterics, residential environment, cooking behaviors, and other sources of household air pollution.

Characteristic	Overall cohort *n* = 116
Maternal characteristics
Age category (years)
18–20	36 (31.0)
21–25	48 (41.4)
≥ 26	32 (27.6)
Parity
Primigravid	73 (62.9)
Secundigravid	43 (37.1)
BMI category (kg/m^2^)
< 18.5	7 (6.0)
18.5–24.9	70 (60.3)
25–29.9	27 (23.2)
≥ 30	12 (10.3)
History of hypertension	5 (4.3)
Treated for antepartum malaria episode	0 (0)
Sociodemographics
Housing
Apartment/multifamily compound	93 (80.5)
Single family home	22 (19.5)
Neighborhood
Urban	41 (35.3)
Periurban or rural	75 (64.7)
Household asset index
Low	6 (5.2)
Medium	62 (53.5)
High	48 (41.4)
Cooking behaviors during measurement
Cooked meals for family	111 (96.5)
Fuels used
Did not cook	4 (3.5)
Wood only	2 (1.7)
Charcoal only	39 (33.9)
Kerosene only	13 (11.3)
Both charcoal and kerosene	55 (47.8)
Gas or electricity	0 (0)
Other mixtures	2 (1.7)
Rainy season during measurement	59 (50.9)
Cooks for commerce	2 (1.7)
Kitchen characteristics
No. of stoves^*a*^	2.0 (0.0)
Cooking area shared with other families	54 (46.6)
Cooking area outdoors/partially outdoors	37 (31.9)
Visible soot on walls	95 (83.3)
Other sources of household air pollution
Use of incense	23 (19.8)
Use of mosquito coils	8 (6.9)
Burning of rubbish	20 (17.4)
Secondhand smoke	17 (14.7)
Tobacco use	3 (2.6)
Nearest road is paved	34 (30.9)
Values are *n* (%) unless otherwise noted. Numbers may not add to sample size because of missing values. ^***a***^Median (interquartile range).	

### Exposure Levels

Among the 79 subjects with PM_2.5_ measurements, the geometric mean personal PM_2.5_ exposure was 40.5 μg/m^3^ (geometric standard deviation: 17.3) with a range from 14.9 to 528.2 μg/m^3^. The first tertile of exposure included exposures ≤ 32.9 μg/m^3^, the second tertile from 33 to 45.6 μg/m^3^, and the upper tertile > 45.6 μg/m^3^. The geometric mean personal CO exposure for the 116 women with these measurements was 2.21 ppm (geometric standard deviation: 1.47) and ranged from 0.34 to 25.15 ppm. The first tertile included exposures ≤ 1.42 ppm, the second tertile from 1.43 to 3.06 ppm, and the upper tertile > 3.06 ppm. As with baseline characteristics, exposure measurements did not differ significantly among subjects with available placental slides compared with the larger group of 239 subjects (data not shown). The strength of correlation between PM_2.5_ and CO measurements for an individual subject was only weakly positive (Pearson’s *r* = 0.30, *p* = 0.008 after ln-transformation), so we continued with separate analyses for PM_2.5_ and CO.

### Association of Exposure with Placental Pathology

***Inflammatory lesions.*** We did not observe a statistically significant association between inflammatory lesions and PM_2.5_ (Table 3), although there was an increased prevalence of inflammatory placental lesions across tertiles of PM_2.5_ (1st tertile, 11.1%; 2nd tertile, 15.4%; 3rd tertile, 26.9%; *p* = 0.15 for trend). The prevalence of chronic villitis (Figure 1), independent of the remainder of the inflammatory lesions, also varied by PM_2.5_ tertile but similarly did not reach statistical significance (1st tertile, 7.4%; 2nd tertile, 11.5%; 3rd tertile, 19.2%; *p* = 0.22 for trend). No association was discernible for CO exposure and inflammatory placental lesions (*p* = 0.42 for trend) (Table 4). Similarly, no association was observed between inflammatory placental lesions and either PM_2.5_ or CO when exposure was considered a continuous predictor after ln-transformation in adjusted logistic models (Tables 3 and 4). Of note, our cohort was recruited from a placental malaria study, and placental malaria is associated with inflammatory placental changes, specifically chronic villitis. However, placental malaria was rare in our cohort (4 of 116, 3.4%). No active malaria was detected, only the presence of malaria pigments indicative of chronic malaria. Furthermore, none of these four cases demonstrated chronic villitis, intervillositis, or other inflammatory lesions.

**Table 3:** Placental pathology by particulate matter exposure.

Placental pathology	PM_2.5_ exposure [*n* (%)]				Adjusted OR^*b*^ (95% CI)
	1st Tertile *n* = 27	2nd Tertile *n* = 26	3rd Tertile *n* = 26	Significance^*a*^	
Placental lesion categories
Hypoxic	7 (25.9)	6 (23.1)	8 (30.8)	0.69	1.4 (0.5, 4.0)
Ischemic/hypertensive	1 (3.7)	4 (15.4)	2 (7.7)	0.64	0.7 (0.1, 6.0)
Inflammatory	3 (11.1)	4 (15.4)	7 (26.9)	0.15	1.8 (0.6, 5.6)
Infectious	7 (25.9)	4 (15.4)	3 (11.5)	0.21	0.1 (0.0, 0.7)
Thrombotic (maternal)	3 (11.1)	7 (26.9)	3 (11.5)	1.00	2.5 (0.7, 8.6)
Thrombotic (fetal)	1 (3.7)	5 (19.2)	4 (15.4)	0.22	5.5 (1.1, 26.8)
Placental weight
Small for gestational age	9 (39.1)	10 (50.0)	10 (55.6)	0.29	1.5 (0.5, 4.8)
Large for gestational age	2 (8.7)	0 (0.0)	2 (11.1)	1.00	2.01 (0.3, 12.7)
Abbreviations: CI, confidence interval; OR, odds ratio; PM_2.5_, fine particulate matter ≤ 2.5 μm. ^***a***^Cochran Armitage Trend test, two-sided *p*-values. Exact *p*-values reported when cell counts < 5. ^***b***^The odds ratios represent the odds of having a placental lesion in the considered category (e.g., hypoxic) for a 1-unit increase in PM_2.5_ exposure on the ln-scale adjusted for age, body mass index, secondhand smoke exposure, season of exposure measurement (rainy vs. dry), and a household asset index.					

**Table 4:** Placental pathology by carbon monoxide exposure.

Placental pathology	CO exposure [*n* (%)]				Adjusted OR^*b*^ (95% CI)
	1st Tertile *n* = 39	2nd Tertile *n* = 39	3rd Tertile *n* = 38	Significance^*a*^	
Placental lesion categories
Hypoxic	8 (20.5)	9 (23.2)	8 (21.1)	0.95	1.0 (0.5, 1.9)
Ischemic/hypertensive	5 (12.8)	3 (7.7)	4 (10.5)	0.73	1.0 (0.4, 2.6)
Inflammatory	5 (12.8)	10 (25.6)	6 (15.8)	0.73	1.4 (0.6, 3.0)
Infectious	9 (23.1)	9 (23.1)	7 (18.4)	0.62	0.7 (0.3, 1.6)
Thrombotic (maternal)	8 (20.5)	5 (12.8)	6 (15.8)	0.57	0.8 (0.4, 1.9)
Thrombotic (fetal)	2 (5.1)	3 (7.7)	8 (21.1)	0.03	2.5 (1.0, 6.4)
Placental weight
Small for gestational age	10 (33.3)	10 (30.3)	15 (46.9)	0.26	1.2 (0.6, 2.4)
Large for gestational age	5 (16.7)	5 (15.2)	3 (9.4)	0.47	0.7 (0.2, 2.0)
Abbreviations: CI, confidence interval; CO, carbon monoxide; OR, odds ratio. ^***a***^Cochran Armitage Trend test, two-sided *p*-values. Exact p-values reported when cell counts < 5. ^***b***^The odds ratios represent the odds of having a placental lesion in the considered category (e.g., hypoxic) for a 1-unit increase in CO exposure on the ln-scale adjusted for age, body mass index, secondhand smoke exposure, season of exposure measurement (rainy vs. dry), and a household asset index.					

***Hypoxic lesions.*** Hypoxic placental lesions were common in the overall cohort (25 of 116, 21.5%); however, the frequency of hypoxic lesions did not vary by either PM_2.5_ or CO exposure (*p* = 0.41 and 0.53, respectively) (Tables 3 and 4). Analysis of chorangiosis or edema separately, rather than together under the broader category of hypoxic lesions, did not alter our findings. Similarly, no association was observed between hypoxic placental lesions and either PM_2.5_ or CO when exposure was considered a continuous predictor after ln-transformation in adjusted logistic models (Tables 3 and 4).

**Figure 3:**
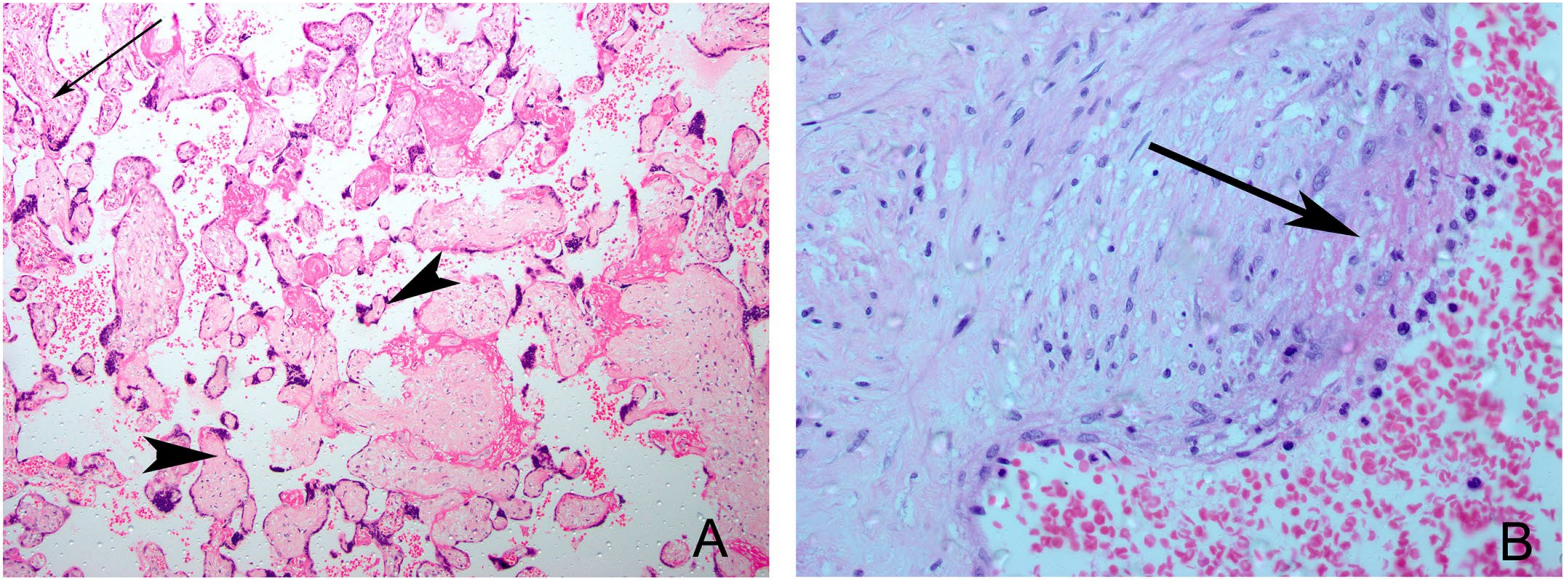
Representative samples of fetal thrombotic vasculopathy. (*A*) Hematoxylin/eosin-stained section of placenta showing normally vascularized villi (arrow) and adjacent field of avascular villi, distal vessel–fetal thrombotic vasculopathy (arrowheads). Magnification: 20×. (*B*) Hematoxylin/eosin-stained section of a stem villous vessel showing an endothelial cushion with a cap of fibrin clot, large vessel–fetal thrombotic vasculopathy, arrow. Magnification: 40×.

***Other placental pathology.*** We identified associations between fetal thrombotic vasculopathy (FTV) (Figure 3) and both PM_2.5_ and CO. The adjusted odds of FTV were 5.5 times higher (95% CI: 1.1, 26.8) per 1-unit increase in the ln-transformed PM_2.5_ measurements after adjustment; notably, the unadjusted effect size was lower and did not reach statistical significance (OR = 2.8; 95% CI: 0.9, 8.9) (see Table S1). Although the trend test across tertiles of PM_2.5_ did not reach statistical significance (*p* = 0.22), the prevalence of FTV was much lower in the first tertile of exposure (3.7%) than in the upper two tertiles (19.2% and 15.4%) (Table 3). For CO, we observed a trend across tertiles of exposure (1st tertile, 5.1%; 2nd tertile, 7.7%; 3rd tertile, 21.1%; *p* = 0.03) (Table 4) and an increase in the odds of diagnosing FTV [adjusted odds ratio (aOR) = 2.5, 95% CI: 1.0, 6.4] with increasing ln-transformed CO exposure after covariate adjustment, although the latter did not quite reach statistical significance in adjusted or unadjusted models (see Table S2). A pattern in the severity or location (large vessel versus distal vessel) of the fetal thrombotic lesions by tertile of PM_2.5_ or CO exposure was not discernible.

We also observed an inverse association between PM_2.5_ and infectious placental lesions when considering PM_2.5_ as a continuous predictor (Table 3). This was driven by a reduction in the prevalence of chorioamnionitis across tertiles, as we observed chronic malaria (pigment) in only four subjects, two of whom had no available PM_2.5_ measurements and the other two with measurements in the uppermost tertile. No other trends were observed for either PM_2.5_ or CO exposure, including no identified associations with small or large for gestational age placental weights. In sensitivity analysis, neither fuel type (kerosene versus charcoal) nor season was associated with any of the considered placental lesion categories (data not shown).

### Association of Placental Pathology with Adverse Birth Outcomes

We explored associations between placental lesion categories and both low birth weight (< 2,500 g) and stillbirth. Preterm birth (< 37 weeks at delivery) was not considered because only 2 of the 116 in our cohort were identified as delivering a preterm infant. Information on preeclampsia was not routinely available from facility records, and study nurses did not obtain blood pressure measurements at delivery. Stillbirth was diagnosed in 5 pregnancies (4.3%) and low birth weight in 13 pregnancies (11.2%). A placenta with FTV was associated with an increased odds of both low birth weight (unadjusted OR = 4.59; 95% CI: 1.2, 17.9) and stillbirth (unadjusted OR = 6.7; 95% CI: 1.0, 44.7). None of the other placental lesion categories were associated with either low birth weight or stillbirth except for placentas demonstrating hypertensive/ischemic lesions which were associated with stillbirth.

## Discussion

With this investigation, we used placental pathology to further evaluate the observed epidemiologic associations of household air pollution with adverse pregnancy outcomes and explore potential mechanisms underlying these effects. We did not identify a statistically significant association between PM_2.5_ and placental inflammation or between CO exposure and placental hypoxia as hypothesized. Instead, we observed that thrombotic placental lesions were linked with both PM_2.5_ and CO exposures in our cohort. The association of household air pollutant exposure with the thrombotic lesions we detected was limited to the fetal circulation of the placenta, manifest as FTV, whereas maternal thrombotic lesions did not vary by exposure to either pollutant. It is possible that the fetus and/or placenta may be particularly vulnerable to household air pollution exposures. Although most placental pathologic findings in the general population are not quantified, because most placentas do not receive a pathologic examination in the absence of pregnancy complications, one study reported the incidence of fetal thrombotic lesions to be 3% in 169 consecutively examined placentas (Vern et al. 2000). In comparison, the frequency with which we observed FTV in the uppermost tertile of both PM_2.5_ and CO exposure was remarkably high (15.4% and 21.1% respectively).

We also observed an increased risk for low birth weight and stillbirth in pregnancies complicated by placental FTV. Prior literature has similarly linked FTV with adverse perinatal outcomes including oligohydramnios, growth restriction, stillbirth, as well as coagulopathy and systemic thrombosis in the newborn (Chisholm and Heerema-McKenney 2015; Saleemuddin et al. 2010). Additionally, severe FTV has been connected with intracranial hemorrhage and neurologic impairment of the infant (Chisholm and Heerema-McKenney 2015). To date, the impact of prenatal exposure to household air pollution on the neurocognitive development of the infant has been understudied although preliminary work suggests adverse effects (Dix-Cooper et al. 2012). We propose this should be an area of intensified study. An additional benefit to be achieved through improvements in household air pollution may be reducing harms to the developing fetal brain.

The strength of this study lies in access to both personal exposure measurements during pregnancy and placental pathology. Measured exposures to PM_2.5_ in our urban/periurban population using predominantly charcoal and kerosene for cooking were lower than exposures reported from rural populations using mostly wood fuel (Dionisio et al. 2012; Jiang and Bell 2008; Smith et al. 2010; Van Vliet et al. 2013). Exposure to carbon monoxide was also lower for our subjects than for rural biomass users, as with PM_2.5_, but the differences were less pronounced than for PM_2.5_ (geometric mean of 2.21 ppm in our study vs. 2.38 ppm for women cooking on open fires in rural Guatemala) (Naeher et al. 2001). That said, the exposures we observed were still moderate to high, and the majority of the women (70/79, 88.6%) exceeded the World Health Organization guideline for acceptable air quality (mean 24-hr PM_2.5_ not to exceed 25 μg/m^3^) (WHO 2010). The lowest exposure tertiles are therefore not “unexposed.” In addition, we measured exposure only once during the latter half of pregnancy, which may not fully characterize gestational exposure. Exposure earlier in pregnancy might be more closely related to placental injury because this period is when trophoblasts transform uterine spiral arteries and establish uteroplacental blood flow (Pijnenborg et al. 1980).

An additional limitation is that we measured exposure to fine particles with a diameter ≤ 2.5 μm. It may be that ultrafine particles, with diameters ≤ 100 nm, are the more relevant exposure for the placenta; our study was not designed to assess this. For inflammation in the placenta to be linked with inhaled exposure to particulate matter, particles must migrate across alveolar spaces into the systemic circulation, leading to direct toxicity of distal tissues (e.g., placenta) or localized inflammation in lung tissues, leading to systemic inflammation. Similar hypotheses have been advanced to explain the pathophysiology underlying the relationship between ambient air pollution, particulate matter, and cardiovascular health (Brunekreef and Holgate 2002; Donaldson et al. 2005). Experiments exposing rats via inhalation to radiolabeled ultrafine particles have demonstrated that particles can be found outside of pulmonary tissues such as in the liver and spleen, although excretion by the kidneys was notably rapid (Kreyling et al. 2002). Particle composition may also be critical to toxicity (Lippmann et al. 2013), and we did not specifically characterize the chemical composition of the fine particles we measured. These limitations may have contributed to the lack of formal statistical significance between exposure and inflammatory or hypoxic placental lesions as initially hypothesized.

A link between personal measures of exposure to household air pollution and fetal placental thrombosis, although not originally hypothesized, is plausible given interconnections between inflammatory reactions and the clotting cascade. Coagulation, inhibition of fibrinolysis, and platelet aggregation have been demonstrated following exposure to particulate matter in both *in vitro* laboratory investigations and animal models (Gilmour et al. 2005; Nemmar et al. 2002). We formally tested a number of hypotheses evaluating associations of both PM_2.5_ and CO with each of the various placental lesion categories, and did not adjust for multiple comparisons. That we identified the same lesion, FTV, for both PM_2.5_ and CO and in both categorical and continuous analyses does strengthen our findings. That said, the exploratory nature of our work, the wide confidence intervals in effect sizes, and the lack of consistency between categorical and continuous outcomes or between adjusted and unadjusted analyses underscores the need for further investigation in larger studies.

## Conclusions

In summary, our results suggest that prenatal exposure to inhaled particulate matter and carbon monoxide may be associated with fetal thrombosis in a dose-dependent matter. Our findings should be verified in larger studies with populations using a variety of biomass fuels in both rural and urban settings. Attention should be paid to obtaining repeated measures of exposure over the course of gestation. FTV may contribute to unfavorable perinatal outcomes such as low birth weight and stillbirth, outcomes that have been associated with household air pollution exposure in pregnancy. Moreover, given a recognized link between fetal thrombosis and adverse neurocognitive development, our findings suggest that additional long-term benefits should be evaluated following reductions in prenatal household air pollution exposure.

## Supplemental Material

(168 KB) PDF
