# Supplementary material for: Placental Pathology Associated with Household Air Pollution in a Cohort of Pregnant Women from Dar es Salaam, Tanzania
Source: Environ Health Perspect. 2016 Jun 10;125(1):134–40. doi: 10.1289/EHP256 (PMC5226703; doi:10.1289/EHP256)
Supplement: (168 KB) PDF [file EHP256.s001.acco.pdf]

*Environ Health Perspect*
DOI: 10.1289/EHP256

**Note to readers with disabilities:** *EHP* strives to ensure that all journal content is accessible to all readers. However, some figures and Supplemental Material published in *EHP* articles may not conform to 508 standards due to the complexity of the information being presented. If you need assistance accessing journal content, please contact ehp508@niehs.nih.gov. Our staff will work with you to assess and meet your accessibility needs within 3 working days.

# Supplemental Material

# Placental Pathology Associated with Household Air Pollution in a Cohort of Pregnant Women from Dar es Salaam, Tanzania

Blair J. Wylie, Emmanuel Matechi, Yahya Kishashu, Wafaie Fawzi, Zul Premji, Brent A. Coull, Russ Hauser, Majid Ezzati, and Drucilla Roberts

## Table of Contents

Details of carbon monoxide exposure measurement
Details of fine particulate matter exposure measurement
**Figure S1**: Identification of the study cohort. Of the 239 subjects enrolled into the primary study on household air pollution among pregnant women in Dar es Salaam, Tanzania, 123 were excluded secondary to a lack of available placental slides. Of the remaining 116, all had available CO measurements. Only 79 of the 116 subjects had available and valid $PM_{2.5}$ measurements. The reasons for excluding $PM_{2.5}$ measurements from the other 37 subjects included: 1 inadequate sampling time, 1 misplaced filters, 4 pump errors, and 31 with implausible weights secondary to incorrect pump setup by one field worker.
**Table S1:** Placental pathology by particulate matter exposure, unadjusted and adjusted models
**Table S2:** Placental pathology by carbon monoxide exposure, unadjusted and adjusted models
References

Details of carbon monoxide exposure measurement

Maternal exposure to carbon monoxide was measured over 72 hours using Draeger Carbon Monoxide 50/a-D (cumulative CO exposure 50-600 ppm-h) passive diffusion tubes (Draeger USA) attached to the mother with a lanyard clipped to her clothing near her breathing zone. Length of color change in the dosimeter tube was measured with metric rulers in the field at 24 hour intervals by research staff until completion of the 72 hour exposure period. A third-order polynomial was fit to the millimeter measurements corresponding to preprinted ppm-h markings on the batch of CO tubes used in the study ($y = -0.006x^3 + 1.0075x^2 + 5.1726x - 0.3053$; y = cumulative exposure in ppm-h and x = dosimeter tube color change in mm), allowing for conversion of color change length in millimeters to exposure in ppm-h. The cumulative exposure in ppm-h was converted to average exposure in ppm through division by the duration of measurement in hours. If available, mean exposure over 72-hrs was used. In the absence of a valid 72-hr measurement, then a 48-hr measurement was used if available and valid. The lower limit of detection (LOD) for the tubes was 1 mm of measured color change over 48 or 72 hours, equivalent to 0.12 or 0.08 ppm respectively. This method has been previously validated in field settings (Smith et al., 2010; Edwards et al., 2007; Dionisio et al., 2012).

Details of fine particulate matter exposure measurement

$PM_{2.5}$ exposure measurements occurred during the first and third 24 hours of the CO measurement using a portable, battery operated Casella Apex Lite Personal Sampling Pump (Casella USA). Particulate matter was collected onto 37mm Teflon membrane filters (Pall Life Sciences: Teflo, 0.2-mm pore size) in conductive polypropylene cassettes (SKC Inc (SKC Inc.) using a GK2.05SH KTL cyclone (BGI by Mesa Labs) with a 50% cut point of 2.5 micrometers at 3.5 liters per minute (LPM) (±10%). The pump was programmed to run one minute out of every four minutes as battery life would not sustain a full 24 hours of operation; the pump therefore ran a total of 6 hours during each 24 hour period. Measurements were not obtained during the second day as the battery required recharging. The cyclone inlet was clipped near the maternal breathing zone, while the sampling pump was worn by the subjects in either a small fanny pack or over the shoulder purse based on subject preference.

$PM_{2.5}$ mass concentrations were measured on a Mettler Toledo MT5 microbalance at the Harvard School of Public Health Laboratory, after being conditioned in a temperature and humidity controlled environment (20.5±0.2ºC, 39±2% relative humidity) for at least 24 hours and statically discharged via a polonium source. In both pre- and post-weighing, filters were weighed twice; if these two masses were not within 5 μg of one another, they were weighed a third time. The mean of the two masses within 5 μg of one another was used for calculating concentrations. Final filter weights were adjusted using an air buoyancy correction (Schoonover and Jones 1981). All filter weights took into account correction for lab blanks. Field blanks were not used.

The average of the two 24-hr $PM_{2.5}$ mass concentrations was used to represent the personal particulate matter exposure of the subject over the sampling period. Samples were excluded from analysis if the sampling duration was less than 80% of planned, if there was a broken connection between the pump and the inlet for a significant period of time leading to an implausible mass, or if a pump error was noted.

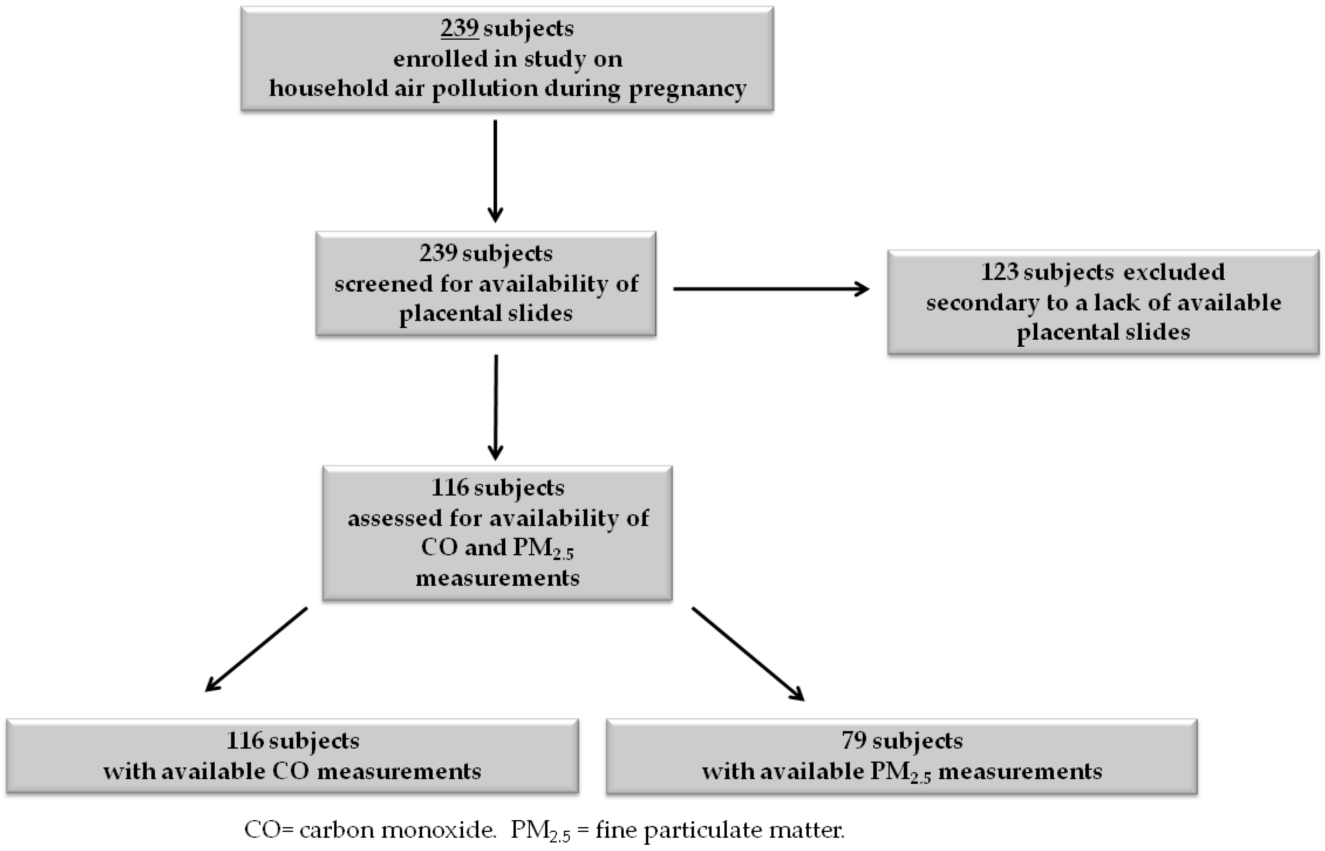


Figure S1: Identification of the study cohort. Of the 239 subjects enrolled into the primary study on household air pollution among pregnant women in Dar es Salaam, Tanzania, 123 were excluded secondary to a lack of available placental slides. Of the remaining 116, all had available CO measurements. Only 79 of the 116 subjects had available and valid $PM_{2.5}$ measurements. The reasons for excluding $PM_{2.5}$ measurements from the other 37 subjects included: 1 inadequate sampling time, 1 misplaced filters, 4 pump errors, and 31 with implausible weights secondary to incorrect pump setup by one field worker.

**Table S1** Placental pathology by particulate matter exposure, unadjusted and adjusted models

| | Unadjusted OR[a] (95% CI) | Adjusted OR[b] (95% CI) |
|---|---|---|
| Placental lesion categories | | |
| Hypoxic | 1.5 (0.6, 3.8) | 1.4 (0.5, 4.0) |
| Ischemic/ Hypertensive | 1.1 (0.2, 4.8) | 0.7 (0.1, 6.0) |
| Inflammatory | 1.5 (0.5, 4.3) | 1.8 (0.6, 5.6) |
| Infectious | 0.1 (0.0, 0.7) | 0.1 (0.0, 0.7) |
| Thrombotic (maternal) | 2.3 (0.8, 6.9) | 2.5 (0.7, 8.6) |
| Thrombotic (fetal) | 2.8 (0.9, 8.9) | 5.5 (1.1, 26.8) |
| Placental Weight | | |
| Small for gestational age | 1.5 (0.6, 4.0) | 1.5 (0.5, 4.8) |
| Large for gestational age | 1.8 (0.4, 7.8) | 2.0 (0.3, 12.7) |

CI= confidence interval. OR= odds ratio. $PM_{2.5}$ = fine particulate matter < 2.5 micrometers.

[a] The odds ratios represent the odds of having a placental lesion in the considered category (e.g., hypoxic) for a one unit increase in $PM_{2.5}$ exposure on the *ln*-scale.

[b]Adjusted for age, body mass index, second hand smoke exposure, season of exposure measurement (rainy vs dry), and a household asset index.

**Table S2** Placental pathology by carbon monoxide exposure, unadjusted and adjusted models

| | | |
|---|---|---|
| | Unadjusted OR (95% CI) | Adjusted OR[b] (95% CI) |
| Placental lesion categories | | |
| Hypoxic | 1.1 (0.5, 2.1) | 1.0 (0.5, 1.9) |
| Ischemic/ Hypertensive | 1.4 (0.6, 3.3) | 1.0 (0.4, 2.6) |
| Inflammatory | 1.3 (0.6, 2.7) | 1.4 (0.6, 3.0) |
| Infectious | 0.8 (0.4, 1.7) | 0.7 (0.3, 1.6) |
| Thrombotic (maternal) | 0.7 (0.3, 1.5) | 0.8 (0.4, 1.9) |
| Thrombotic (fetal) | 2.5 (1.0, 6.3) | 2.5 (1.0, 6.4) |
| Placental weight | | |
| Small for gestational age | 1.2 (0.6, 2.3) | 1.2 (0.6, 2.4) |
| Large for gestational age | 0.8 (0.3, 1.9) | 0.7 (0.2, 2.0) |

CI= confidence interval. CO= carbon monoxide. OR= odds ratio.

[a] The odds ratios represent the odds of having a placental lesion in the considered category (e.g., hypoxic) for a one unit increase in $PM_{2.5}$ exposure on the *ln*-scale.

[b]Adjusted for age, body mass index, second hand smoke exposure, season of exposure measurement (rainy vs dry), and a household asset index.

.

## REFERENCES

Dionisio KL, Howie SRC, Dominici F, Fornace KM, Spengler JD, Adegbola RA, et al. 2012. The exposure of infants and children to carbon monoxide from biomass fuels in The Gambia: a measurement and modeling study. J Expo Sci Environ Epidemiol 22:173-181.

Edwards RD, Liu Y, He G, Yin Z, Sinton J, Peabody J, et al. 2007. Household CO and PM measured as part of a review of China's National Improved Stove Program. Indoor Air 17(3):189-203.

Schoonover R, Jones F. 1981. Air buoyancy correction in high-accuracy weighing on analytical balances. Anal Chem 53(6):900-902.

Smith KR, McCracken JP, Thompson L, Edward R, Shields KN, Canuz E, et al. 2010. Personal child and mother carbon monoxide exposures and kitchen levels: methods and results from a randomized trial of woodfired chimney cookstoves in Guatemala (RESPIRE). J Expo Sci Environ Epidemiol 20(5):406-416.
